# The Health Physician

**Published:** 1889-12

**Authors:** 


### THE HEALTH PHYSICIAN.

NEXT month it will be the duty of the Board of Health to appoint the Health Physician of this city for the ensuing year. Without disparagement to any other worthy candidate, we favor the reappointment of Dr. Edward Clark, the present incumbent, because we believe he has shown himself thoroughly competent not only to fulfil the ordinary requirements of the office, but to rise equal to great emergencies and subdue impending epidemics. We have only to remind the profession, and the citizens generally, of the threatened small-pox invasion in the Summer of 1888, which was prevented from being a serious calamity to the city through the energetic and capable management of Dr. Clark. He devoted himself, day and night, to the task of limiting the outbreak to the quarter of the city where it first appeared, and was so successful that, notwithstanding the alarm felt at such times, we are assured that no business interest suffered in the least, and the great International Fair was able to be opened at the appointed time. Much more could be said on this subject, especially in regard to the slight cost to the city, as compared with what other cities have had to expend on similar occasions; but the events are of such recent occurrence it does not seem necessary.

Dr. Clark has also interested the authorities in the effort to obtain a pure food supply, or rather to prevent diseased products from being sold as food, and has thus inaugurated an important work. We believe that public officers who prove themselves capable should be retained in the public service as long as they can be, and we trust the Board of Health will appreciate the importance of applying this principle to the office in question.

